# Etiologic subtypes of first and recurrent ischemic stroke in young patients using A-S-C-O and TOAST classification criteria: A retrospective follow-up study

**DOI:** 10.1177/23969873241238508

**Published:** 2024-03-25

**Authors:** Karoliina Aarnio, Nicolas Martinez-Majander, Elena Haapaniemi, Eeva Kokkola, Jenna Broman, Lauri Tulkki, Markku Kaste, Turgut Tatlisumak, Jukka Putaala

**Affiliations:** 1 Department of Neurology, University of Helsinki and Helsinki University Hospital, Helsinki, Finland; 2 Sahlgrenska Academy, University of Gothenburg & Department of Neurology, Sahlgrenska University Hospital, Gothenburg, Sweden

**Keywords:** Ischemic stroke, young adults, etiology, risk factors, recurrent stroke

## Abstract

**Introduction:**

Scarce data exist on the etiology of recurrent ischemic strokes (ISs) among young adults. We analyzed the etiology of first-ever and recurrent events and the differences between them.

**Patients and methods:**

Patients aged 15–49 years with a first-ever IS in 1994–2007 were included in the Helsinki Young Stroke Registry. In this retrospective cohort study, data on recurrent ISs were identified from Care Register for Health Care until the end of 2017 and Causes of Death Register and from patient records until the end of 2020. All first-ever and recurrent ISs were classified using Atherosclerosis-Small vessel disease-Cardioembolism-Other Cause (A-S-C-O) and Trial of Org 10172 in Acute Stroke Treatment (TOAST) classifications.

**Results:**

A total of 970 patients were included (median age at index IS 46 years, interquartile range 43–48, 33% women), of which 155 (16.0%) patients had recurrent IS, with 8 (5.2%) fatal cases and 5 (3.2%) unverifiable cases. The median follow-up was 17.4 (IQR 13.9–21.7) years. Median time from the index event to the first recurrent event was 4.5 (interquartile range [IQR] 1.6–10.2) years. Recurrence was more often due to definite cardioembolism (10.7% vs 18.0%, *p* = 0.013), while the proportion of other definite A-S-C-O subgroups remained the same. With TOAST classification, the proportion of true cryptogenic ISs decreased (16.7% vs 6.7%, *p* = 0.003), while those with incomplete evaluation increased (9.3% vs 19.3%, *p* = 0.015). Other TOAST phenotypes remained the same.

**Conclusion:**

The proportion of definite cardioembolism increased at recurrence using the A-S-C-O classification and the number of cryptogenic ISs decreased using the TOAST classification, while cases with incomplete evaluation increased. Most etiologies remained the same.

## Graphical abstract

**Graphical abstract:**
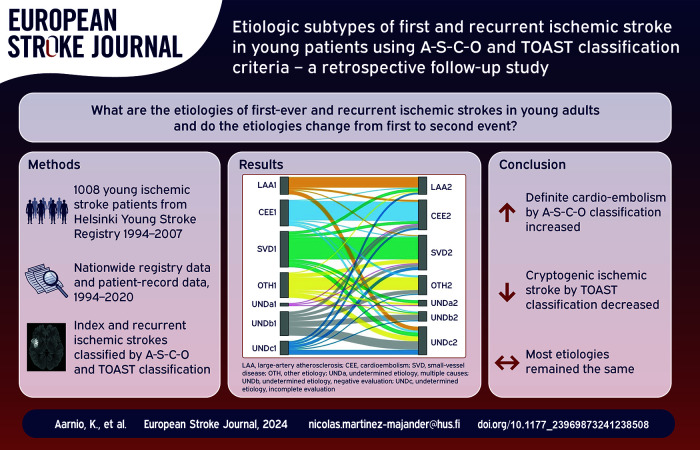

## Introduction

Ischemic stroke (IS) is one of the leading causes of death and disability worldwide.^1^ Around 10% of all ISs affect young adults, who typically have several years of active life ahead of them and family and work to attend.^2^ Therefore, their recurrent vascular events and long-term outcome after IS are important, but still understudied.

The etiology of young-onset IS differs from that of the elderly.^3^ Young-onset ISs are more frequently caused by less well-established mechanisms, such as cervical artery dissection, or more strongly associated with findings, such as patent foramen ovale (PFO), compared to older adults with more ISs associated with large-artery atherosclerosis (LAA) and small vessel disease (SVD).^4^ However, the most widely used etiological classification Trial of Org 10172 in Acute Stroke Treatment (TOAST) does not take into account several less well-established etiologies nor does it consider the probability of the etiology.^5^ TOAST classification also lacks detailed criteria for the etiologic work-up. A prospective multicenter study on young-onset IS using a newer A-S-C-O classification (A for atherosclerosis, S for SVD, C for cardiac source, O for other cause)^6^ showed that many young patients presenting with acute IS had concomitant stroke etiologies associated with substantial atherosclerotic risk profile.^7^

Although there are some studies reporting the etiologies of recurrent ISs in older patients, little is known about the etiologies of stroke recurrence among younger adults.^8^ One recent study exploring recurrent IS after young-onset embolic stroke of undetermined source (ESUS) found that a substantial number, as much as 68%, of recurrent ISs could also be classified as ESUS.^9^ Also, up to 30% of the etiologies of recurrent IS remained undetermined despite comprehensive and timely diagnostic work-up. However, another study reported that even in young-onset cryptogenic ISs, some risk factors and triggers were still present, including vigorous physical exercise and fever.^10^

The aim of this study was to analyze the etiology of the index and recurrent IS in young adults from the Helsinki Young Stroke Registry (HYSR) using both the A-S-C-O and TOAST criteria, and to explore whether the etiology differs between the index and recurrent event.

## Patients and methods

### Study population

The HYSR covers all consecutive patients aged 15–49 years with first-ever IS from January 1994 to May 2007 selected from a prospective electronic hospital discharge database at the Department of Neurology, Helsinki University Hospital (HUH), which has the only 24/7 neurological emergency unit for a population of 1.5 million.^4^ We used the World Health Organization definition of stroke with the exception that patients with imaging-positive findings in the territory corresponding to clinical presentation were classified as an IS even if the symptoms lasted less than 24 h.

Diagnostic tests have already been previously described.^4^ All patients underwent brain computed tomography (CT) or magnetic resonance imaging (MRI). The size of the infarct was determined as the size of the largest ischemic lesion based on documented criteria as previously described.^11^ The use of secondary preventive medication at 3 months after the index event is based on information received from the patient over the phone and patient medical records. Modified Rankin Scale (mRS) score was used to depict the functional outcome of patients at 3 months after the index and recurrent event.^12^ We retrospectively classified the causes of stroke using the A-S-C-O and TOAST classifications based on medical records and available imaging and laboratory data.^5,6^

We excluded patients who could not be linked to any of the databases needed to obtain outcome data, patients with a false primary diagnosis, patients with no medical records available of the index/recurrent IS, and those who died within 30 days of the index IS.

### Follow-up data

We obtained data on hospitalizations until the end of 2017 from the Care Register for Health Care of the National Institute for Health and Welfare, Finland, and data on dates and primary and contributory causes of death as International Classification of Diseases (ICD)-codes from the Statistics Finland until the end of 2020. The Care Register for Health Care includes all patients hospitalized in Finland since 1994, and reporting is mandatory for all public healthcare delivery facilities.^13^ The Causes of Death Registry maintained by Statistics Finland covers all deaths and causes of death of Finnish citizens reported by treating physicians using ICD codes since 1936.^14^ We included all IS-related hospitalizations with discharge diagnosis codes for primary or secondary cause of hospitalization using the ICD 9th Revision 1994–1995 (434, 436, 437) and the 10th Revision from 1996 onwards (I63, I64, I67). In addition, we verified and performed a search of patient records for recurrent events not found from the registry and until the end of 2020 for all included patients. The follow-up period started from the index IS and ended on December 31, 2020, or the date of moving outside the HUH catchment area, or the date of death, whichever occurred first.

### Recurrent stroke

E.H., K.A., and N.M.M. verified all first non-fatal and fatal recurrent ISs from medical records where possible. In discrepant cases a consensus was reached by J.P., K.A., and N.M.M. We defined recurrent ISs as a rapid onset of a new persistent neurological deficit caused by cerebrovascular obstruction without an apparent non-vascular cause verified by CT or MRI.^15^ We excluded events that were considered to be transient ischemic attacks (TIA) if symptoms lasted less than 24 h and no imaging findings of acute infarction were found. However, imaging-positive events were considered as recurrent ISs despite the short duration of symptoms. The patient characteristics were as defined at the index event. The use of secondary preventive medications at the time of recurrent IS is based solely on patient records.

### A-S-C-O and TOAST modified classifications

All index and recurrent events were classified by A-S-C-O and TOAST classifications (E.H., K.A., N.M.M). The A-S-C-O classifications of those with recurrent events and those with discrepancies between A-S-C-O and TOAST classifications based on clinical, radiographical and laboratory data were cross-checked by another investigator (K.A. or N.M.M.). At admission of the index event, all patients underwent routinely a range of blood tests, chest x-ray, electrocardiogram (ECG), and brain imaging, as well as other previously described tests.^4^ The A-S-C-O classifications were based on the predefined minimum number of investigations.^6^ For the TOAST classifications of recurrent ISs, the minimum requirements included brain CT or MRI, cervical vessel imaging by ultrasound, CT or MR angiography, basic laboratory tests, ECG, and echocardiogram, to be classified as TOAST 5b, negative evaluation, if these investigations yielded negative results.

The A-S-C-O classification provides three grades of evidence/likelihood: 1) definitely a potential cause of IS (1), 2) causality uncertain (2), 3) unlikely a direct cause of IS but disease is present (3); and 4) insufficient work-up (9). Post-radiation angiopathy was considered as A-S-C-O O=1. To better adapt to young stroke, we also applied a modified TOAST criteria by dividing TOAST 2 into high-risk causes of cardioembolism (CE high-risk) and low-risk causes of cardioembolism (CE low-risk), the latter comprising PFO and/or atrial septal aneurysm (ASA), wall hypokinesia/akinesia/history of myocardial infarction (MI) and other/undetermined valve disease.^16^ Also, both carotid and vertebral dissections were reported separately from the other etiology group, TOAST 4, as subgroups. TOAST 1 (LAA), TOAST 2 (SVD), and TOAST 5 (5a two or more causes identified, 5b negative evaluation, and 5c incomplete evaluation) were left unchanged. The Risk of Paradoxical Embolism (RoPE) scores were calculated for all PFO-related strokes.^17^

### Standard protocol approvals, registrations, and patient consents

We coded all data anonymously into the study database. A regional Ethics Committee approved the study protocol. According to Finnish legislation, a written informed consent is waived in registry-based studies.

### Statistical analyses

We analyzed data using IBM SPSS Statistics, Version 25 for Windows (IBM, Armonk, NY) and with R Studio. The characteristics of included patients were presented with descriptive statistics (numbers and percentages; medians and interquartile ranges [IQRs]). For comparison of independent samples, we used Pearson’s chi-squared test and Mann-Whitney U test. We used McNemar’s test to compare the difference between binary index and recurrent stroke characteristics, as well as A-S-C-O and TOAST categories within classes, Wilcoxon signed rank test for non-normally distributed continuous variables, and Marginal homogeneity (SPSS 2-related samples) test for the total categorical comparison for TOAST and A-S-C-O classes. We used the Sankey diagram to depict the change of etiologies from the index to the recurrent event. *p* < 0.05 were considered significant.

## Results

### Characteristics at baseline and at recurrence

**Figure 1.**
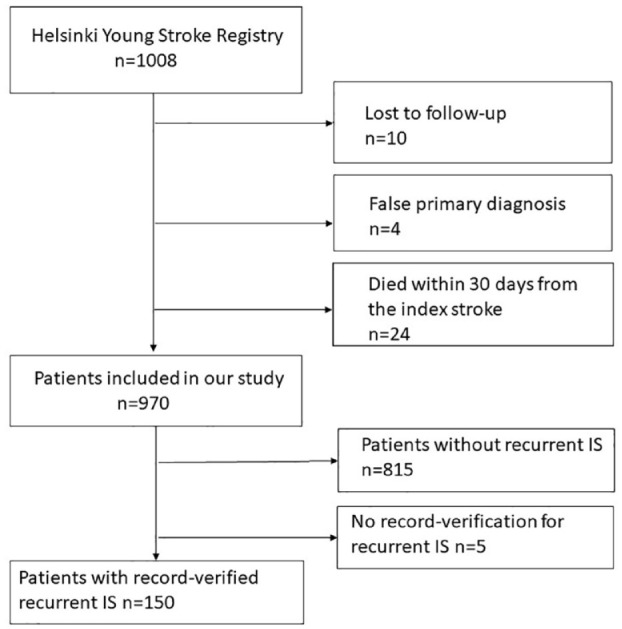
Flow chart of the study population. IS: ischemic stroke.

Figure 1 describes patients included in the final analyses. Of the 1008 patients in the HYSR, a total of 970 patients were included (median age at first-ever IS onset 46 years, interquartile range 43–48, 33% women). Of these, 155 (16.0%) patients had recurrent IS, of which data from 5 (3.2%) patients could not be verified, which resulted in 150 patients with complete etiological classifications. A search for recurrent events from medical records yielded in five new first recurrent ISs not recorded in the Hospital Discharge or Death Registries and seven new cases between 2018 and 2020, when registry data for hospitalizations were lacking. The median follow-up was 17.4 (IQR 13.9–21.7) years. The median time from the index event to the first recurrent IS was 4.5 (1.6–10.2) years. Comparison of baseline characteristics at the index IS showed that patients without recurrent IS were younger, less likely to be current smokers, and less likely to have hypertension, any type of diabetes, or peripheral artery disease. At 3 months, they were more often on anticoagulants but less likely to use antiplatelets or antihypertensives (Table S1).

Of these 150 verified and imaging-positive patients, 8 (5.2%) had fatal recurrent events, and 107 (71.3%) had imaging-positive lesions. A head CT was performed for 144 (96.0%) patients and/or a brain MRI for 63 (42.0%) patients with recurrent ISs. Initially, 9 (6.0%) recurrent ISs using the TOAST classification and 12 (8.0%) recurrent ISs using the ASCO classification were differently classified by two investigators. In these cases, the investigators reached a consensus.

In the 150 patients with recurrent IS, the overall number of risk factors per patient shown in Table 1 increased significantly between the index and recurrent IS (2 [1–3], vs 3 [2–3], *p* < 0.001). At recurrence, patients had more frequently hypertension (72.7% vs 50.7%, *p* < 0.001), type 2 diabetes mellitus (21.3% vs 12.0%, *p* = 0.001), atrial fibrillation (10.0% vs 4.7%, *p* = 0.008), coronary heart disease (13.3% vs 6.0%, *p* = 0.003), prior MI (8.0% vs 3.3%, *p* = 0.039), and peripheral artery disease (10.0% vs 4.0%, *p* = 0.022) compared to index IS. In contrast, current cigarette smoking decreased (54.0% vs 39.3%, *p* < 0.001). Furthermore, recovery from the recurrent IS was poorer compared to index IS (median mRS 2 [IQR 1–3] vs 2 [IQR 1–2], *p* < 0.001). There were no differences in other risk factors, infarct location or size, or in admission National Institutes of Health Stroke Scale (NIHSS) score. A significant proportion of patients had discontinued antiplatelet (77.0% vs 65.3%, *p* = 0.012) and anticoagulant (23.0% vs 15.3%, *p* = 0.035) therapy between the 3 months after the index IS and recurrent IS, while the use of statins and antihypertensives was more frequent at the time of recurrent IS.

**Table 1.** Risk factors and stroke features in patients with recurrent IS (*n* = 150) at index and recurrent event.

	Index IS	Recurrent IS	*p*-Value
Patient characteristics			
Age, years	46 (43–48)	51 (47–55)	<0.001
Male	100 (66.7)	100 (66.7)	1.000
Cardiovascular risk factors			
Atrial fibrillation	7 (4.7)	15 (10)	0.008
Cigarette smoking	81 (54.0)	59 (39.3)	<0.001
Congestive heart failure	10 (6.7)	16 (10.7)	0.180
Coronary heart disease	9 (6.0)	20 (13.3)	0.003
Dyslipidemia	101 (67.3)	107 (71.3)^a^	0.349
Hypertension	76 (50.7)	109 (72.7)	<0.001
Myocardial infarction	5 (3.3)	12 (8.0)	0.039
Type 1 diabetes mellitus	14 (9.3)	15 (10.0)	1.000
Type 2 diabetes mellitus	18 (12.0)	32 (21.3)	0.001
Peripheral artery disease	6 (4.0)	15 (10.0)	0.022
No. of risk factors	2 (1–3)	3 (2–3)	<0.001
Stroke characteristics			
Infarct size			0.179
Small	75 (50.0)	90 (60.0)^b^	
Medium	40 (26.7)	38 (25.3)^b^	
Large anterior	23 (15.3)	14 (9.4)^b^	
Large posterior	12 (8.0)	7 (4.7)^b^	
Stroke severity (NIHSS at admission)	3 (2–6)	3 (2–7)	0.471
mRS at 3 months	2 (1–2)	2 (1–3)^c^	<0.001
Medications in use at 3 months after index event/at recurrency			
Antiplatelets	114 (77.0)^a^	98 (65.3)	0.012
Anticoagulants	34 (23.0)^a^	23 (15.3)	0.035
Antihypertensives	71 (47.3)	95 (63.3)	<0.001
Statins	40 (26.8)^b^	58 (38.7)	0.012

Data are expressed as median (interquartile range) or *n* (%).

IS: ischemic stroke; NIHSS: National Institute of Health Stroke Scale; mRS: modified Rankin Scale.

a Data missing for two patients.

b Data missing for one patient.

c Data missing for 19 patients.

### Etiology between index and recurrent ischemic stroke using the TOAST classification

**Figure 2.**
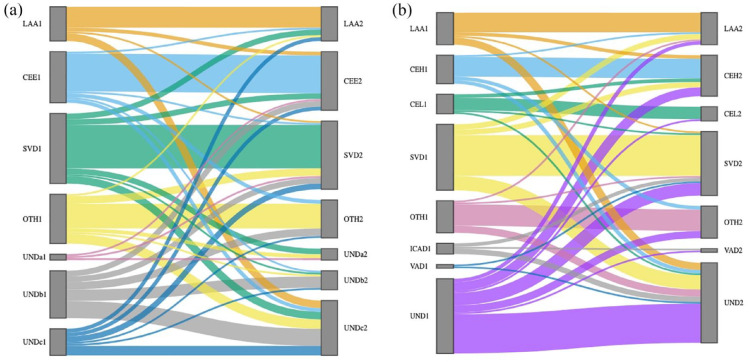
The change of etiology from the index to the recurrent event in (a) TOAST and (b) modified TOAST classification system by Sankey diagram. LAA: large-artery atherosclerosis; CEH: high-risk sources of cardioembolism; CEL: low-risk sources of cardioembolism; SVD: small vessel disease; OTH: other etiology than dissection; ICAD: internal carotid artery dissection; VAD: vertebral artery dissection; UND: undetermined etiology.

Classified with TOAST, the proportion of true cryptogenic ISs decreased (16.7% vs 6.7%, *p* = 0.003), while the proportion of patients with incomplete evaluation (5c) increased (9.3% vs 19.3%, *p* = 0.015) between the index and recurrent IS. The proportion of other TOAST phenotypes 1–3 remained the same (Table 2, Figure 2(a)). The etiologies of index and recurrent IS were exactly the same in 11 of 18 (61.1%) index ISs caused by LAA, 20 of 27 (74.1%) caused by cardioembolism, 23 of 37 (62.2%) caused by SVD, 13 of 26 (50.0%) caused by other causes, 1 of 3 (33.3%) caused by multiple possible etiologies, 6 of 25 (24.0%) index cryptogenic ISs, and 5 of 14 (35.7%) ISs with incomplete evaluation. PFO alone was diagnosed in 8 (5.3%) patients both at index and at recurrent IS. The median RoPE Score was 8.0 (IQR 7.0–8.3) at index versus 6.0 (IQR 4.3–7.8) at recurrent event. Furthermore, at index IS, 2 (1.3%) patients had PFO with ASA.

**Table 2.** Etiologies of IS at index and recurrent event by modified TOAST classification (*n* = 150).

Modified TOAST classification	Index IS	Recurrent IS	*p*-Value
LAA	18 (12.0)	18 (12.0)	1.000
CE high-risk	16 (10.7)	23 (15.3)	0.143
CE low-risk	11 (7.3)	8 (5.3)	0.375
SVD	37 (24.7)	36 (24.0)	1.000
Other than dissection	18 (12.0)	18 (12.0)	1.000
ICAD	6 (4.0)	0 (0.0)	0.031
VAD	2 (1.3)	2 (1.3)	1.000
UND			
Two or more causes identified	3 (2.0)	6 (4.0)	0.453
Negative evaluation	25 (16.7)	10 (6.7)	0.003
Incomplete evaluation	14 (9.3)	29 (19.3)	0.015

Data are expressed as *n* (%).

Marginal homogeneity test for the whole modified TOAST versus recurrent modified TOAST nonsignificant (*p* = 0.565).

TOAST: Trial of Org 10172 in Acute Stroke Treatment; IS: ischemic stroke; LAA: large artery atherosclerosis; CE: cardioembolism; SVD: small vessel disease; ICAD: internal carotid artery dissection; VAD: vertebral artery dissection; UND: undetermined etiology.

### Etiology between index and recurrent ischemic stroke using the modified TOAST classification

Using the modified TOAST classification, the prevalence of ISs caused by internal carotid artery dissection (ICAD) decreased (4.0% vs 0.0%, *p* = 0.031) while it remained unchanged for ISs caused by vertebral artery dissection (VAD) or ISs caused by other defined etiologies (Table 2, Figure 2(b)). Similarly, the prevalence of CE high-risk and CE low-risk remained the same (10.7% vs 15.3%, *p* = 0.143 and 7.3% vs 5.3%, *p* = 0.375, respectively). Of the 25 patients with an index cryptogenic IS (TOAST 5b), 1 had a recurrent IS due to vertebral artery dissection, 1 due to low-risk causes of CE, namely PFO, 2 due to high-risk causes of CE, 3 due to SVD, 3 due to other causes than dissection, namely 2 due to vasculitis and 1 due to APC resistance, 9 had incomplete evaluation, and 6 remained cryptogenic despite routine examinations.

In the 6 (5.2%) patients with fatal record-verified recurrences, 3 (50.0%) were due to high-risk sources of cardioembolism, 2 (33.3%) due to LAA and 1 (16.7%) due to other causes than dissection. Furthermore, of the 150 patients with a recurrent IS, multiple recurrences were verified in 26 (17.3%) patients (median 2, IQR 2–3). The majority of the second recurrences (*n* = 26) were undetermined ISs (34.6%) or ISs caused by rare causes other than dissection (30.8%). These rare syndromes included cerebral autosomal dominant arteriopathy with subcortical infarcts and leukoencephalopathy (CADASIL, *n* = 2), primary angiitis of the central nervous system (PACNS, *n* = 3), one patient with secondary central nervous system vasculitis associated with systemic lupus erythematosus (SLE), one patient with severe anemia and hypovolemia, and one patient with polycytemia vera, heterozygous Factor V Leiden mutation and warfarin below therapeutic level. Eight patients experienced up to three recurrent ISs during the follow-up, five of which were due to a rare cause: one CADASIL, two PACNS, one vasculitis associated with SLE, and one patient with a thrombosed basilar artery aneurysm.

Finally, in the 815 patients with no recurrent IS, the most frequent etiology was undetermined (34.7%), followed by SVD (12.5%), other than dissection (10.6%), VAD (10.1%), CE low-risk (9.9%), CE high-risk (8.8%), ICAD (7.9%), and LAA (5.5%).

### Etiology between index and recurrent ischemic stroke using the A-S-C-O classification

Table 3 shows the overall distribution of grade of certainty for each etiological subgroup according to A-S-C-O in both index and recurrent IS. Definitely a potential cause (A-S-C-O grade 1) could be verified in 76 index ISs, compared to 89 recurrences. At the time of the index IS, atherosclerosis was the most frequent definite cause (A = 1, 13.3%), followed by SVD (S = 1, 12.7%). In contrast, in recurrent ISs, definite SVD (S = 1, 18%) and cardioembolism (C = 1, 18%) were the largest groups, while atherosclerosis remained unchanged (A = 1, 13.3%). Recurrent events were more often due to definite cardioembolism compared to index ISs (18.0% vs 10.7%, *p* = 0.013) (Table 4). Compared to patients with recurrent IS, in the 815 patients with no recurrence, the most frequent definite etiology was other defined cause (O = 1, 23.1%), followed by cardioembolism (C = 1, 8.8%), SVD (S = 1, 6.3%), and atherosclerosis (A = 1, 5.8%).

**Table 3.** Etiologies of IS at index and recurrent event by A-S-C-O classification (*n* = 150).

Index IS					
Grade of certainty	A	S	C	O	All
0 = disease absent	103	89	59	25	276
1 = definitely a potential cause	20	19	16	21	76
2 = causality uncertain	2	28	4	1	35
3 = unlikely a direct cause	17	14	15	7	53
9 = insufficient work-up	8	0	56	96	160
Recurrent IS					
Grade of certainty	A	S	C	O	All
0 = disease absent	60	87	37	5	189
1 = definitely a potential cause	20	27	26	16	89
2 = causality uncertain	2	20	4	3	29
3 = unlikely a direct cause	39	16	10	5	70
9 = insufficient work-up	29	0	73	121	223

IS: ischemic stroke; A: atherosclerosis; S: small vessel disease; C: cardioembolism; O: other reason.

**Table 4.** Comparison of definite A-S-C-O between index and recurrent event.

A-S-C-O	Index IS	Recurrent IS	*p*-Value
A1 definite	20	20	1.000
S1 definite	19	27	0.115
C1 definite	16	27	0.013
O1 definite	21	16	0.302

IS: ischemic stroke; A: atherosclerosis; S: small vessel disease; C: cardioembolism; O: other reason.

## Discussion

In our retrospective cohort study, we found that in a significant number of 150 recurrent ISs in young adults, the etiologies remained mostly the same as in the index event. Up to 17% of patients with recurrent ISs experienced more than one recurrent event. Only the number of definitive cardioembolic strokes increased and the number of cryptogenic strokes decreased, however, including more cases with incomplete diagnostic work-up at the time of recurrence.

To our knowledge, this is the first study among young stroke patients to investigate differences in the etiologies of index and recurrent ISs. Among an older patient population looking at etiologies using the A-S-C-O classification, definite cardioembolic strokes were also more frequent at the recurrent event.^8^ In that study, the etiological work-up at recurrency, especially regarding cardiological investigations, were less frequent, tallying with our observations.^8^

In a recent meta-analysis on recurrence of IS involving patients over 18 years of age, recurrence rates were higher with LAA and cardioembolic strokes, where the etiology remained mostly the same as in recurrency.^18^ On the contrary, the meta-analysis found that among ISs caused by SVD, the etiology in recurrent IS was more versatile.^18^ In our study, most patients with LAA IS, cardioembolic and SVD infarcts had the same etiology at recurrence, while cryptogenic strokes decreased, and incomplete diagnostic work-up increased at recurrence.

Also, compliance with secondary preventive medication seems to be poor in the long run, with less than 40% of patients using statins at recurrence, although over 70% had dyslipidemia. The same trend applies to antihypertensives, indicating the need for better secondary preventive medication strategies and follow-up. According to our previous findings in a shorter follow-up, the use of antihypertensives was suboptimal in one third of patients in whom antihypertensives were initially prescribed.^19^ Also, less than half of young IS patients used statins at end of follow-up in 2012 (vs ischemic stroke 1994–2007).^20^ Similarly, the use of antiplatelets (65.3%) and anticoagulants (15.3%) was low at recurrency in the longer follow-up, possibly due to poor compliance, but hypothetically it could also be due to, for instance, hemorrhagic complications, and thus the patients’ inability to use medications as prescribed. Recurrent ISs should be avoided, especially since the mRS was higher after recurrent events than the mRS after the index event, leading to greater residual symptoms.

Both the A-S-C-O and TOAST classifications have weaknesses, but a comprehensive classification system for IS is not yet available. A-S-C-O has relatively strict and specific rules for classification for the required etiological work-up.^6^ However, during the years, etiological work-up schemes have evolved and, for instance, prolonged rhythm monitoring to screen for atrial fibrillation is performed more systematically than in the past.^21^ It can also be debated whether lumbar puncture is necessary for all patients with otherwise unknown etiology in A-S-C-O classification, as this is not done in routine practice unless an inflammatory etiology is clinically suspected. Similarly, the most widely used TOAST classification has its weaknesses. It does not give detailed requirements on investigations needed for specific etiologies, nor does it indicate if there are multiple etiologies, the etiologies found. Also, for instance, the classification of PFO-related strokes is not given in detail.

Our study has limitations. First, due to the long period of inclusion of patients, the etiologic work-up protocols have varied over time, and due to the retrospective study design, protocols have varied between patients. Then again, this better reflects the real-life situation. Also, for instance, the introduction of direct oral anticoagulants for non-valvular atrial fibrillation has changed treatment strategies in stroke patients. Second, with more advanced imaging techniques, such as high-resolution vessel wall imaging, for example, the proportion of undetermined IS and SVD might decrease and the proportion of LAA might increase, as was the case in a recent Chinese study.^22^ Also, there might still be unidentified etiologies constituting now the undetermined group, such as the newly suggested clonal hematopoiesis of indetermined potential (CHIP).^23^ Cancer screening was rarely done routinely, only in cases with high clinical suspicion, although it is a possible etiology of IS.^24^ Third, A-S-C-O-D is a newer classification system that takes into account arterial dissection as a separate group, but also includes other changes compared to the previous A-S-C-O classification system.^25^ As our cohort study already used the older A-S-C-O classification, we did not switch to A-S-C-O-D but considered vertebral and carotid arterial dissections in the modified TOAST classification as separate groups. Similarly, for PFO-related ISs, we only graded the RoPE score but did not have all the information needed for doing the newer and more extensive PFO-Associated Stroke Causal Likelihood grading.^26^ We were unable to report in more detail whether the diagnostic work-up for recurrent IS actually had an impact on secondary prevention or outcome. Finally, some very early recurrences may have been missed because those who died within 30 days were excluded.

Nevertheless, there are strengths in our study. We have a large cohort consisting of solely young IS patients with a long follow-up time and a significant number of recurrent events. The coverage (90%), sensitivity (95%–97%) and positive predictive value (87%–93%) of stroke diagnoses have been rated as good in the Care Register for Health Care, from which the recurrent ISs were primarily retrieved.^27^ We had the possibility to verify the etiologies from original patient records and not only rely on ICD codes or registry data. The TOAST and A-S-C-O classifications can be difficult to use, and the inter-rater agreement rate is not always good enough from a clinical point of view, although in one study it was rated high,^28^ which led us to double-check the classifications and reach a consensus in difficult cases.

## Conclusions

Our study showed that most etiologies remained unchanged in the index and recurrent IS also among young adults. In the future, prospective studies are needed to systematically determine the etiologies of recurrent ISs. Our study also emphasizes that etiological work-up should be systematically repeated also in recurrent ISs to avoid mistargeting of tertiary prevention efforts.

## Supplemental Material

sj-docx-1-eso-10.1177_23969873241238508 – Supplemental material for Etiologic subtypes of first and recurrent ischemic stroke in young patients using A-S-C-O and TOAST classification criteria: A retrospective follow-up study Supplemental material, sj-docx-1-eso-10.1177_23969873241238508 for Etiologic subtypes of first and recurrent ischemic stroke in young patients using A-S-C-O and TOAST classification criteria: A retrospective follow-up study by Karoliina Aarnio, Nicolas Martinez-Majander, Elena Haapaniemi, Eeva Kokkola, Jenna Broman, Lauri Tulkki, Markku Kaste, Turgut Tatlisumak and Jukka Putaala in European Stroke Journal

## References

[1] GBD 2019 Stroke Collaborators. Global, regional, and national burden of stroke and its risk factors, 1990-2019: a systematic analysis for the Global Burden of Disease Study 2019. Lancet Neurol 2021; 20: 795–820. 34487721 10.1016/S1474-4422(21)00252-0 PMC8443449

[2] Aarnio K Rodríguez-Pardo J Siegerink B , et al. Return to work after ischemic stroke in young adults: A registry-based follow-up study. Neurology 2018; 91: e1909–e1917. 10.1212/WNL.0000000000006510 PMC6260196 30315074

[3] Ekker MS Boot EM Singhal AB , et al. Epidemiology, aetiology, and management of ischaemic stroke in young adults. Lancet Neurol 2018; 17: 790–801. 30129475 10.1016/S1474-4422(18)30233-3

[4] Putaala J Metso AJ Metso TM , et al. Analysis of 1008 consecutive patients aged 15 to 49 with first-ever ischemic stroke: the Helsinki young stroke registry. Stroke 2009; 40: 1195–1203. 19246709 10.1161/STROKEAHA.108.529883

[5] Adams HP Bendixen BH Kappelle LJ , et al. Classification of subtype of acute ischemic stroke. Definitions for use in a multicenter clinical trial. Toast. Trial of org 10172 in acute stroke treatment. Stroke 1993; 24: 35–41. 7678184 10.1161/01.str.24.1.35

[6] Amarenco P Bogousslavsky J Caplan LR , et al. New approach to stroke subtyping: the A-S-C-O (phenotypic) classification of stroke. Cerebrovasc Dis 2009; 27: 502–508. 19342826 10.1159/000210433

[7] Wolf ME Grittner U Böttcher T , et al.. Phenotypic ASCO characterisation of young patients with ischemic stroke in the prospective multicentre observational sifap1 study. Cerebrovasc Dis 2015; 40: 129–135. 26227782 10.1159/000434760

[8] Wolf ME Sauer T Hennerici MG , et al. Characterization of patients with recurrent ischaemic stroke using the ASCO classification. Eur J Neurol 2013; 20: 812–817. 23293855 10.1111/ene.12068

[9] Perera K de Sa Boasquevisque D Rao-Melacini P , et al. Evaluating rates of recurrent ischemic stroke among young adults with embolic stroke of undetermined source: the Young ESUS longitudinal cohort study. JAMA Neurol 2022; 79: 450–458. 35285869 10.1001/jamaneurol.2022.0048 PMC8922202

[10] Ekker MS Verhoeven JI Schellekens MMI , et al. Risk factors and causes of ischemic stroke in 1322 young adults. Stroke 2023; 54: 439–447. 36511150 10.1161/STROKEAHA.122.040524 PMC9855752

[11] Aarnio K Joensuu H Haapaniemi E , et al. Cancer in young adults with ischemic stroke. Stroke 2015; 46: 1601–1606. 25922510 10.1161/STROKEAHA.115.008694

[12] van Swieten JC Koudstaal PJ Visser MC , et al. Interobserver agreement for the assessment of handicap in stroke patients. Stroke 1988; 19: 604–607. 3363593 10.1161/01.str.19.5.604

[13] Finnish Institute for Health and Welfare. Care Register for Health Care, Accessed 21 Jan 2024, https://thl.fi/en/statistics-and-data/data-and-services/register-descriptions/care-register-for-health-care

[14] Official Statistics of Finland (OSF). Causes of death [e-publication]. Helsinki: Statistics Finland. Accessed 21 Jan 2024, http://www.stat.fi/til/ksyyt/index_en.html

[15] Sacco RL Kasner SE Broderick JP , et al. An updated definition of stroke for the 21st century: a statement for healthcare professionals from the American Heart Association/American Stroke Association. Stroke 2013; 44: 2064–2089. 23652265 10.1161/STR.0b013e318296aeca PMC11078537

[16] Schnabel RB Camen S Knebel F , et al. Expert opinion paper on cardiac imaging after ischemic stroke. Clin Res Cardiol 2021; 110: 938–958. 34143285 10.1007/s00392-021-01834-x PMC8238761

[17] Kent DM Ruthazer R Weimar C , et al. An index to identify stroke-related vs incidental patent foramen ovale in cryptogenic stroke. Neurology 2013; 81: 619–625. 23864310 10.1212/WNL.0b013e3182a08d59 PMC3775694

[18] Kolmos M Christoffersen L Kruuse C. Recurrent ischemic stroke – a systematic review and meta-analysis. J Stroke Cerebrovasc Dis 2021; 30. 105935. 10.1016/j.jstrokecerebrovasdis.2021.105935 34153594

[19] van Dongen MME Aarnio K Martinez-Majander N , et al. Use of antihypertensive medication after ischemic stroke in young adults and its association with long-term outcome. Ann Med 2019; 51: 68–77. 30592437 10.1080/07853890.2018.1564358 PMC7857461

[20] van Dongen MME Aarnio K Martinez-Majander N , et al. Use of statins after ischemic stroke in young adults and its association with long-term outcome. Stroke 2019; 50: 3385–3392. 31699020 10.1161/STROKEAHA.119.026992

[21] Roy AT Schwamm LH Singhal AB. Use of prolonged cardiac rhythm monitoring to identify atrial fibrillation after cryptogenic stroke. Curr Cardiol Rep 2022; 24: 337–346. 35171442 10.1007/s11886-022-01652-1

[22] He WW Lu SS Ge S , et al. Impact on etiology diagnosis by high-resolution vessel wall imaging in young adults with ischemic stroke or transient ischemic attack. Neuroradiol 2023; 65: 1015–1023. 10.1007/s00234-023-03131-y 36806972

[23] Mayerhofer E Strecker C Becker H , et al. Prevalence and therapeutic implications of lonal hematopoiesis of indeterminate potential in young patients with stroke. Stroke 2023; 54: 938–946. 36789775 10.1161/STROKEAHA.122.041416 PMC10050122

[24] Dardiotis E Aloizou AM Markoula S , et al. Cancer-associated stroke: Pathophysiology, detection and management (review). Int J Oncol 2019; 54: 779–796. 30628661 10.3892/ijo.2019.4669 PMC6365034

[25] Amarenco P Bogousslavsky J Caplan LR , et al. The ASCOD phenotyping of ischemic stroke (updated ASCO phenotyping). Cerebrovasc Dis 2013; 36: 1–5. 10.1159/000352050 23899749

[26] Elgendy AY Saver JL Amin Z , et al. Proposal for updated nomenclature and classification of potential causative mechanism in patent foramen ovale–associated stroke. JAMA Neurol 2020; 77: 878–886. 32282016 10.1001/jamaneurol.2020.0458

[27] Sund R. Quality of the Finnish Hospital Discharge Register: a systematic review. Scand J Public Health 2012; 40: 505–515. 22899561 10.1177/1403494812456637

[28] Wolf ME Sauer T Alonso A , et al. Comparison of the new ASCO classification with the TOAST classification in a population with acute ischemic stroke. J Neurol 2012; 259: 1284–1289. 22146904 10.1007/s00415-011-6325-1

