# Supplementary material for: Etiologic subtypes of first and recurrent ischemic stroke in young patients using A-S-C-O and TOAST classification criteria: A retrospective follow-up study
Source: Eur Stroke J. 2024 Mar 25;9(4):1034–42. doi: 10.1177/23969873241238508 (PMC11569552; doi:10.1177/23969873241238508)
Supplement: sj-docx-1-eso-10.1177_23969873241238508 – Supplemental material for Etiologic subtypes of first and recurrent ischemic stroke in young patients using A-S-C-O and TOAST classification criteria: A retrospective follow-up study [file sj-docx-1-eso-10.1177_23969873241238508.docx]

**SUPPLEMENTAL MATERIAL**

**Supplemental Table 1.**

**Table S1**. Comparison of baseline characteristics in patients without recurrent ischemic stroke (n=815) and those with recurrent ischemic stroke (n=150).

|  | **Patients without recurrent IS** | **Patients with recurrent IS** | **P-value** |
| --- | --- | --- | --- |
| **Patient characteristics** |  |  |  |
| Age, years | 43 (37-47) | 46 (43-48) | <0.001 |
| Male | 506 (62.1) | 100 (66.7) | 0.286 |
| **Cardiovascular risk factors** |  |  |  |
| Atrial fibrillation | 31 (3.8) | 7 (4.7) | 0.617 |
| Cigarette smoking | 350 (42.9) | 81 (54.0) | 0.012 |
| Congestive heart failure | 35 (4.3) | 10 (6.7) | 0.205 |
| Coronary heart disease | 36 (4.4) | 9 (6.0) | 0.398 |
| Dyslipidemia | 482 (59.1) | 101 (67.3) | 0.059 |
| Hypertension | 306 (37.5) | 76 (50.7) | 0.003 |
| Myocardial infarction | 26 (3.2) | 5 (3.3) | 0.927 |
| Type 1 diabetes mellitus | 28 (3.4) | 14 (9.3) | 0.001 |
| Type 2 diabetes mellitus | 40 (4.9) | 18 (12.0) | <0.001 |
| Peripheral artery disease | 11 (1.3) | 6 (4.0) | 0.023 |
| No. of risk factors | 2 (1-2) | 2 (1-3) | <0.001 |
| **Stroke characteristics** |  |  |  |
| Infarct size |  |  | 0.264 |
| Small | 354 (43.4) | 75 (50.0) |  |
| Medium | 232 (28.5) | 40 (26.7) |  |
| Large anterior | 123 (15.1) | 23 (15.3) |  |
| Large posterior | 106 (13.0) | 12 (8.0) |  |
| Stroke severity (NIHSS at admission) | 3 (1-6) | 3 (2-6) | 0.451 |
| mRS at 3 months | 1 (0-2) | 2 (1-2) | 0.445 |
| **Medications in use at 3 months after index event*** |  |  |  |
| Antiplatelets | 519 (64.3) | 114 (77.0) | 0.003 |
| Anticoagulants | 287 (35.5) | 34 (23.0) | 0.003 |
| Antihypertensives | 282 (34.6) | 71 (47.3) | 0.003 |
| Statins | 203 (25.1) | 40 (26.8) | 0.657 |

Data are expressed as median (interquartile range) or n (%).

IS, ischemic stroke; NIHSS, National Institute of Health Stroke Scale; mRS, modified Rankin Scale.

^*^Data missing for patients without recurrent IS vs with recurrent IS: antiplatelets (8 vs 2), anticoagulants (7 vs 2), antihypertensives (1 vs 0), and statins (7 vs 1).
